# Supplementary material for: Implementing circularity measurements in industry 4.0-based manufacturing metrology using MQTT protocol and Open CV: A case study
Source: PLoS One. 2023 Oct 13;18(10):e0292814. doi: 10.1371/journal.pone.0292814 (PMC10575513; doi:10.1371/journal.pone.0292814)
Supplement: S1 File — (DOCX) [file pone.0292814.s001.docx]

**Appendix A
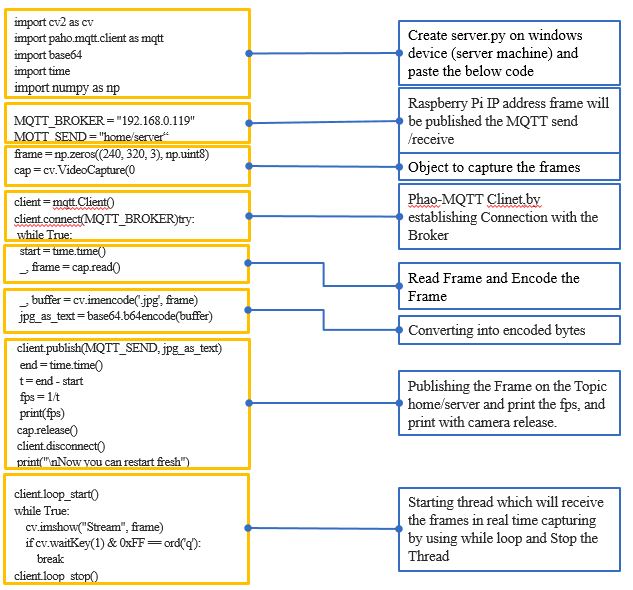
**

**The implemented code of MQTT Protocol**

**Appendix B**

# The full code *# local Yazid saif implemented at AMMC lab (UTHM)*

*# calibration updated regarding the object of interest*

import os, sys, time, traceback

import frame_capture

import frame draw

import cv2

from math import hypot

import numpy as np

frameWidth = 640

frameHeight = 480

cap = cv2.VideoCapture(0)

cap.set(3, frameWidth)

cap.set(4, frameHeight)

def empty(a):

    pass

cal_range = 72

cal = dict([(x,cal_range/dm) for x in range

(0,int(dm)+1,pixel_base)])

cal_base = 5

cal_last = None

*# calibration updated regarding the object of interest*

**def** cal_update(x,y,unit_distance):
    *# basics*

   pixel_distance = hypot(x,y)

   scale = abs(unit_distance/pixel_distance)

 target = baseround(abs(pixel_distance),pixel_base)

low  = target*scale - (cal_base/2)

    high = target*scale + (cal_base/2)

    start = target

    if unit_distance <= cal_base:

        start = 0

    else:

        while start*scale > low:

            start -= pixel_base

    stop = target

    if unit_distance >= baseround(cal_range,pixel_base):

        high = max(cal.keys())

   else:        while stop*scale < high:

            stop += pixel_base

    for x in range(start,stop+1,pixel_base):

    cal[x] = scale

   print(**f**'CAL: {x} {scale}')

cv2.namedWindow("Parameters")

cv2.resizeWindow("Parameters",640,240)

cv2.createTrackbar("Threshold1","Parameters",23,255,empty)

cv2.createTrackbar("Threshold2","Parameters",20,255,empty)

cv2.createTrackbar("Area","Parameters",5000,30000,empty)

def stackImages(scale,imgArray):

    rows = len(imgArray)

    cols = len(imgArray[0])

    rowsAvailable = isinstance(imgArray[0], list)

    width = imgArray[0][0].shape[1]

    height = imgArray[0][0].shape[0]

    if rowsAvailable:

        for x in range ( 0, rows):

            for y in range(0, cols):

                if imgArray[x][y].shape[:2] == imgArray[0][0].shape [:2]:

                    imgArray[x][y] = cv2.resize(imgArray[x][y], (0, 0), None, scale, scale)

                else:

                    imgArray[x][y] = cv2.resize(imgArray[x][y], (imgArray[0][0].shape[1], imgArray[0][0].shape[0]), None, scale, scale)

                if len(imgArray[x][y].shape) == 2: imgArray[x][y]= cv2.cvtColor( imgArray[x][y], cv2.COLOR_GRAY2BGR)

        imageBlank = np.zeros((height, width, 3), np.uint8)

        hor = [imageBlank]*rows

        hor_con = [imageBlank]*rows

        for x in range(0, rows):

            hor[x] = np.hstack(imgArray[x])

        ver = np.vstack(hor)

    else:

        for x in range(0, rows):

            if imgArray[x].shape[:2] == imgArray[0].shape[:2]:

                imgArray[x] = cv2.resize(imgArray[x], (0, 0), None, scale, scale)

            else:

                imgArray[x] = cv2.resize(imgArray[x], (imgArray[0].shape[1], imgArray[0].shape[0]), None,scale, scale)

            if len(imgArray[x].shape) == 2: imgArray[x] = cv2.cvtColor(imgArray[x], cv2.COLOR_GRAY2BGR)

        hor= np.hstack(imgArray)

        ver = hor

    return ver

def getContours(img,imgContour):

    contours, hierarchy = cv2.findContours(img, cv2.RETR_EXTERNAL, cv2.CHAIN_APPROX_NONE)

    for cnt in contours:

        area = cv2.contourArea(cnt)

        areaMin = cv2.getTrackbarPos("Area", "Parameters")

        if area > areaMin:

            cv2.drawContours(imgContour, cnt, -1, (255, 0, 255), 7)

            peri = cv2.arcLength(cnt, True)

            approx = cv2.approxPolyDP(cnt, 0.02 * peri, True)

            print(len(approx))

            x , y , w, h = cv2.boundingRect(approx)

            cv2.rectangle(imgContour, (x , y ), (x + w , y + h ), (0, 255, 0), 5)

            cv2.putText(imgContour, "Points: " + str(len(approx)), (x + w + 20, y + 20), cv2.FONT_HERSHEY_COMPLEX, .7,

                        (0, 255, 0), 2)

            cv2.putText(imgContour, "Area: " + str(int(area)), (x + w + 20, y + 45), cv2.FONT_HERSHEY_COMPLEX, 0.7,

                        (0, 255, 0), 2)

while True:

    success, img = cap.read()

    imgContour = img.copy()

    imgBlur = cv2.GaussianBlur(img, (7, 7), 1)

    imgGray = cv2.cvtColor(imgBlur, cv2.COLOR_BGR2GRAY)

    threshold1 = cv2.getTrackbarPos("Threshold1", "Parameters")

    threshold2 = cv2.getTrackbarPos("Threshold2", "Parameters")

    imgCanny = cv2.Canny(imgGray,threshold1,threshold2)

    kernel = np.ones((5, 5))

    imgDil = cv2.dilate(imgCanny, kernel, iterations=1)

    getContours(imgDil,imgContour)

    imgStack = stackImages(0.8,([img,imgCanny],

                                [imgDil,imgContour]))

    cv2.imshow("Result", imgStack)

    if cv2.waitKey(1) & 0xFF == ord('q'):

        break

**The full Codes of model based mulita algorithms on 3SMVI system**
